# Partial and Total Substitution of Soybean Meal with Black Soldier Fly Larvae Meal in Japanese Quail Diets: Effects on Performance Criteria and Feed Cost Scenarios

**DOI:** 10.3390/ani16030415

**Published:** 2026-01-28

**Authors:** Nihan Öksüz Narinç, Nilgün Yapıcı, Ali Aygun, Doğan Narinç

**Affiliations:** 1 Department of Finance and Banking, Faculty of Applied Sciences, Akdeniz University, Antalya 07070, Turkey; nihanoksuz@akdeniz.edu.tr; 2 Department of Animal Science, Faculty of Agriculture, Akdeniz University, Antalya 07070, Turkey; nilgunyapici@gmail.com; 3 Department of Animal Science, Faculty of Agriculture, Selçuk University, Konya 42100, Turkey; aaygun@selcuk.edu.tr

**Keywords:** black soldier fly larvae meal, alternative protein sources, growth dynamics, feed efficiency, scenario-based economic analysis

## Abstract

The aim of this study was to evaluate the effects of graded replacement of soybean meal (SBM) with black soldier fly larvae meal (BSFLM) on growth performance, growth dynamics, carcass characteristics, and economic efficiency in Japanese quails (*Coturnix japonica*). A total of 300 one-day-old quail chicks were randomly allocated to five dietary treatments in which SBM was replaced with BSFLM at 0, 25, 50, 75, or 100% using isocaloric and isonitrogenous diets. Body weight was recorded weekly, feed intake was measured per cage, and growth dynamics were assessed using the Gompertz growth model. At 42 d of age, 150 quails were slaughtered to determine carcass yield and major carcass components, and economic evaluation was performed using scenario-based analyses to compare feed cost efficiency under contrasting ingredient price conditions. Dietary inclusion of BSFLM had no significant effects on body weight at any measured age, mortality rate, or carcass yield and composition. Feed intake and feed conversion ratio were significantly improved at the 50% BSFLM inclusion level, indicating improved feed efficiency at moderate replacement. Gompertz growth parameters, including mature weight, growth rate, and inflection point traits, were not affected by dietary treatment, confirming that intrinsic growth patterns were maintained. Economic analyses showed that partial replacement of SBM with BSFLM was associated with improved or stabilized feed cost efficiency depending on relative ingredient prices, whereas higher inclusion levels were more sensitive to unfavorable price conditions. In conclusion, BSFLM can be incorporated into Japanese quail diets without detrimental effects on growth performance or carcass traits, with moderate inclusion levels providing the most consistent balance between biological efficiency and economic robustness, thereby supporting risk-aware and sustainable poultry feeding strategies under variable market conditions.

## Simple Summary

This study evaluated the effects of graded replacement of soybean meal with black soldier fly larvae meal in Japanese quail diets on growth performance, carcass traits, and economic efficiency. Replacing soybean meal with black soldier fly larvae meal did not adversely affect body weight, survivability, or carcass characteristics, while moderate inclusion levels improved feed efficiency. Scenario-based economic analyses indicated that these efficiency gains were associated with greater feed cost stability under contrasting ingredient price conditions. Overall, the results show that black soldier fly larvae meal is a nutritionally adequate protein source for quail production, with particular advantages at moderate inclusion levels that support more resilient and sustainable poultry feeding systems.

## 1. Introduction

The sustained growth of the global poultry sector has intensified the demand for nutritionally efficient and economically viable feed ingredients, particularly high-quality protein sources for monogastric species. SBM has long served as the primary dietary protein component in poultry nutrition due to its favorable amino acid profile and high digestibility. However, increasing price volatility, competition with human food and biofuel industries, and environmental concerns associated with soybean cultivation have driven substantial interest in alternative protein sources that align with sustainability and circular-economy principles [1,2]. In this context, insect-derived proteins have emerged as promising candidates for poultry feeding systems. Insects are characterized by high feed conversion efficiency, reduced land and water requirements, and the capacity to valorize low-value organic substrates into nutrient-dense biomass [1]. Among the insect species evaluated for feed purposes, BSFLM have attracted particular attention due to their high crude protein content, favorable essential amino acid composition, and appreciable lipid fraction, which together support their potential as a substitute for conventional protein sources [3,4]. Nevertheless, the nutritional value of BSFLM is not uniform and depends strongly on larval rearing substrate, developmental stage, and post-harvest processing conditions [5,6]. Structural components such as chitin, a polysaccharide inherent to the insect exoskeleton, have been identified as potential constraints at higher dietary inclusion levels due to their effects on nutrient digestibility and intestinal physiology [3,7]. Conversely, moderate chitin intake has also been associated with functional effects on gut health and immune modulation, suggesting a dose-dependent biological response [8].

Experimental evidence in poultry species indicates that BSFLM can be incorporated into diets without detrimental effects on growth performance, feed efficiency, or carcass traits when inclusion levels are properly managed. In broiler chickens, several studies have demonstrated that low to moderate inclusion of BSFLM sustains body weight gain and feed conversion efficiency, whereas responses at higher inclusion levels are more variable and influenced by formulation strategy and ingredient quality [9,10,11]. Recent meta-analytical evaluations further support the notion that BSFLM can effectively replace conventional protein sources within biologically tolerable ranges while emphasizing the importance of inclusion level and processing method in determining performance outcomes [12,13].

Compared with broiler chickens, research on the application of black BSFLM in Japanese quail nutrition remains relatively limited, but available evidence provides important insight into biologically tolerable inclusion ranges. Cullere et al. [14] reported that dietary inclusion of partially defatted BSFLM at levels up to 15% did not impair growth performance, feed intake, or carcass yield in broiler quails, while maintaining acceptable nutrient digestibility and meat quality traits. Similarly, Silva et al. [15] demonstrated that graded replacement of SBM with BSFLM at moderate inclusion levels supported body weight gain and feed efficiency in growing quails, with no adverse effects on survivability or carcass characteristics. These studies collectively indicate that BSFLM can effectively replace conventional protein sources in quail diets when inclusion levels are carefully managed, particularly within low to intermediate substitution ranges. However, existing quail studies have primarily emphasized conventional performance indicators and short-term biological responses, providing limited insight into growth dynamics across the full production period and largely omitting economic evaluations under variable feed-price conditions. Consequently, further evidence integrating growth performance, growth-curve characteristics, and scenario-based economic outcomes is required to support robust biological and economic conclusions under practical feeding conditions.

The aim of this study was to evaluate graded replacement of SBM with BSFLM in Japanese quails by integrating growth performance, carcass traits, and feed cost efficiency, thereby providing a biologically and economically grounded assessment under practical feeding conditions.

## 2. Materials and Methods

This study was conducted at the Animal Husbandry Facilities of the Department of Animal Science at the Faculty of Agriculture at Akdeniz University (Turkey). All the procedures described were approved by Akdeniz University Ethics Committee on Animal Use (protocol number E-74568308-020-637530). The care and management of the birds used in this research were conducted in line with relevant Republic of Turkey laws and regulations.

### 2.1. Experimental Design and Diets

This study’s animal material comprised 300 quail chicks, which were randomly mated and obtained concurrently from a parent flock that had not undergone prior genetic selection. One-day-old chicks were randomly allocated to each experimental group in a completely randomized design. The wing numbers were attached immediately post-hatching, and individual weights were recorded. Weekly live weight measurements were conducted separately using the wing numbers. The chicks were housed in heated broody cages until sex determination was conducted on the 14th day post-hatching. Chicks were accommodated at a stocking density of 20 per cage compartment (90 cm^2^/quail), with three replications in each experimental group [16]. Upon reaching two weeks of age, the quails were relocated to fattening cages, with four quails allocated to each compartment (180 cm^2^/quail). Therefore, the number of replications in each experimental group was augmented to 15. After sex determination at day 14, males and females were redistributed within each dietary treatment so that each compartment contained a balanced number of birds from each sex. Chicks were maintained under standard brooding conditions, with the ambient temperature initially set at 32 °C and gradually reduced by 1 °C every three days to reach 27 °C by the end of the second week, and were provided ad libitum access to feed and water under a 24:0 followed by a 16:8 h light–dark photoperiod.

The BSFL utilized in this study were cultivated at the Faculty of Agriculture, Akdeniz University, using exclusively plant-based food waste as substrate. The substrate consisted of pre-consumer fruit and vegetable processing by-products obtained from a fruit and vegetable processing facility (Organized Industrial Zone, Antalya, Turkey) and included mixed residues such as cabbage, potato, zucchini, carrot, apple, pear, and kiwi. Defatted BSFLM contained 55.6% crude protein, 5.1% crude fat, and 6.2% chitin, as determined by AOAC techniques [5]. In addition, the composition of BSFLM included 6.7% mineral matter, 9.8% ether extract, 12.8% neutral detergent fiber, and 4.9% acid detergent fiber. The experimental diets (Table 1) comprised a basal formula based on maize and soybean, supplemented with BSFLM, designated as BSF0, BSF25, BSF50, BSF75, and BSF100 groups, corresponding to SBM substitutions of 0%, 25%, 50%, 75%, or 100%, respectively. All the diets were formulated to be isocaloric and isonitrogenous. BSF larvae used in the study were reared at Akdeniz University Faculty of Agriculture, and only plant-based food waste was used as substrate. Experimental diets corresponding to each treatment group were provided from day-old chicks (DOC) onward and continued throughout the entire experimental period.

### 2.2. Measurements

Individual live weights were recorded at hatching and subsequently each week until the age of slaughter (42 days). Feed intake was recorded for each cage compartment on the same dates. Dead animals were recorded on a daily basis, and feed efficiency was determined according to the weight of dead birds. Feed efficiency, expressed as feed conversion ratio (FCR), was calculated as the ratio of cumulative feed intake to body weight gain.

The Gompertz growth curve was applied to the growth data of Japanese quails to estimate the relationship between body weight and age [17]. The mathematical representation of the Gompertz function and the coordinates of the inflection point are displayed in Table 2.

In the equation, “t” represents time, “y” signifies weight, “$\beta_{0}$“ indicates the maximum body weight the bird is presumed capable of attaining, “$\beta_{1}$“ symbolizes the biological constant pertaining to the curve’s shape, and “$\beta_{2}$“ refers to the biological constant concerning the growth rate [17].

Thirty randomly selected quails (15 females and 15 males) from each experimental group, totaling 150 quails, were sent for slaughter at 42 days of age. The feed was withheld for six hours prior to slaughter, and the weights of the quails were recorded post-slaughter. All weight measurements during the cutting process were conducted using a digital scale with a precision of 0.01 g. After slaughter, wet plucking, and evisceration, the hot carcass weights were measured, incorporating neck and abdominal fat, while omitting edible internal parts. At this juncture, the weights of edible viscera, including fat in the abdomen, heart, liver, and devoid gizzard, were ascertained. Following a one-day storage of the carcasses at +4 °C, the cold carcass weight was recorded, after which the carcasses were shredded, and the weights of the breast, breast muscle, leg, and wings were measured. By calculating the ratios of cold carcass, edible internal organs, abdominal fat, breast, leg, and wing weights to the slaughter weight, phenotypic values were derived for cold carcass ratio, edible internal organs ratio, abdominal fat ratio, breast ratio, breast muscle ratio, leg ratio, and wing ratio, respectively.

### 2.3. Economy

Economic evaluation was conducted to quantify the feed-cost implications of graded replacement of SBM with BSFLM under contrasting ingredient-price environments. Three price scenarios were defined a priori while keeping the remaining ingredient prices (TL: Turkish Lira) constant across diets, S1 (low-cost BSFLM) SBM = 17.1 TL/kg and BSFLM = 10.6 TL/kg; S2 (medium-cost BSFLM) SBM = 17.1 TL/kg and BSFLM = 16.1 TL/kg; and S3 (high-cost BSFLM) SBM = 17.1 TL/kg and BSFLM = 21.6 TL/kg; the high-cost scenario was derived from the European break-even cost of defatted BSFLM reported (618 €/t DM, converted using the 2024 average EUR/TL rate) by Leipertz et al. [18], and the medium- and low-cost scenarios represent analytically defined parity and lower-bound assumptions reflecting the strong sensitivity of BSFLM costs to production conditions and substrate availability [18,19]. Diet formulations for BSF0, BSF25, BSF50, BSF75, and BSF100 were taken from the experimental rations (Table 1), where SBM and BSFLM inclusion levels were adjusted reciprocally.

For each treatment and scenario, (i) protein meal cost per bird (TL/bird) attributable to SBM + BSFLM was calculated by multiplying cumulative feed intake per bird (FI, kg) by the diet-level cost contribution of SBM and BSFLM (Σ inclusion rate × unit price) [20]. In parallel, (ii) total feed cost per bird (TL/bird) was computed as FI (kg) × total diet cost (TL/kg), where total diet cost was obtained by summing all ingredient inclusion rates (kg/kg) multiplied by their respective unit prices under each scenario. Cumulative feed intake at 42 d (FI42) served as the basis for cost calculations because slaughter occurred at day 42.

To enable biologically anchored interpretation of costs, three complementary economic analyses were implemented:

I. Cost efficiency indices (primary analysis): Total feed cost per bird and feed cost per unit of output, expressed as TL per kg live weight at day 42 (total feed cost ÷ BW42 in kg), and optionally TL per kg gain of initial weight is included (gain-based denominator). BW42 and FI42 used in these indices correspond to the performance outcomes in Table 3.

II. Marginal substitution/break-even analysis: The incremental cost difference (ΔTL/bird) relative to the control diet (BSF0) was computed for each scenario and inclusion level. Additionally, a break-even BSFLM price can be derived (holding SBM price constant) by solving for the BSFLM unit price at which the total feed cost of a given replacement level equals BSF0, thereby identifying the price threshold for economic neutrality.

III. Sensitivity analysis (price-risk analysis): Scenario-based comparisons were interpreted as a discrete sensitivity test to BSFLM price variation under a fixed SBM reference price, allowing assessment of whether biological improvements in feed efficiency (notably at intermediate inclusion) remain economically advantageous as BSFLM prices increase.

### 2.4. Statistical Analyses

Analysis of variance was employed to ascertain the differences between the means of the groups regarding all the obtained characteristics. Mortality data were analyzed using a generalized linear model due to their binomial distribution. The dose–response effects of the inclusion of BSF meal (25, 50, 75, and 100 g/kg) were analyzed by orthogonal contrasts for linear and quadratic effects [15]. If the null hypothesis was rejected, Duncan multiple range test was used. The significance level was established at 0.05 for all statistical analyses.

Gompertz growth model parameters were examined utilizing the NLIN procedure of SAS 9.3 software, employing the Levenberg–Marquardt iteration method [21]. All statistical analyses were performed using SAS software (version 9.3; SAS Institute Inc., Cary, NC, USA).

## 3. Results

### 3.1. Growth Characteristics

The effects of replacing SBM with BSFLM at varying inclusion levels (0%, 25%, 50%, 75%, and 100%) in quail diets on weekly live weight, cumulative feed consumption, cumulative feed conversion ratios, and mortality are presented in Table 3. There were no statistically significant differences among the treatment groups for body weight (BW) at days 28, 35, and 42 (*p* > 0.05). Final BW values at day 42 ranged from 201.39 g to 206.60 g, indicating that dietary BSFLM inclusion did not adversely affect growth performance. Cumulative feed intake (FI) at day 28 did not differ among the groups (*p* > 0.05). However, significant differences were observed in FI at days 35 and 42 (both *p* < 0.05). At both time points, the group receiving 50% BSFLM showed the lowest feed intake, while the BSF0, BSF25, and higher replacement groups had higher values. Cumulative feed conversion ratio (FCR) at day 28 and 35 were not significantly affected by dietary treatments (both *p* > 0.05). Nevertheless, at day 42, FCR showed a significant difference among groups (*p* < 0.05). The BSF50 group had the lowest FCR value (3.13), suggesting improved feed efficiency at this inclusion level. Mortality rates ranged from 3.33% to 11.67% and were not significantly influenced by the dietary treatments (*p* > 0.05). No significant linear or quadratic effects were detected for any performance parameter (*p* > 0.05).

The effects of replacing SBM with BSFLM at different levels (0%, 25%, 50%, 75%, and 100%) on the parameters of the Gompertz growth curve are presented in Table 4. No significant differences were observed among the treatment groups for any of the Gompertz growth curve parameters (*p* > 0.05). The mature weight parameter (β_0_) ranged from 244.93 g to 252.96 g across treatment groups, with no significant linear or quadratic effects determined (both *p* > 0.05). Similarly, the shape parameter (β_1_) and the instantaneous growth rate parameter (β_2_) showed no statistically significant variation among groups (both *p* > 0.05). The body weight at the inflection point (IPW) and the age at the inflection point (IPA) were also not significantly affected by dietary BSFLM inclusion (both *p* > 0.05). The IPW values ranged from 90.11 to 93.07 g, while IPA values ranged from 17.15 to 17.78 days among the groups.

### 3.2. Carcass Characteristics

The effects of replacing SBM with BSFLM at varying levels (0%, 25%, 50%, 75%, and 100%) on slaughter and carcass characteristics are presented in Table 5. No statistically significant differences were observed among the treatment groups for carcass yield, cold carcass weight, breast weight, leg weight, wing weight, or abdominal fat (*p* > 0.05 for all traits). Carcass yield ranged from 69.50% to 69.84%, and cold carcass weight varied between 140.02 g and 144.21 g across all groups. Similarly, breast weights were similar among treatments, ranging from 54.95 g to 56.12 g. Leg weights ranged from 29.93 g to 30.91 g, and wing weights from 11.06 g to 11.69 g, with no significant linear or quadratic trends observed for any of these characteristics. Abdominal fat values ranged between 0.92 g and 1.12 g, and differences among groups were not statistically significant (*p* > 0.05).

### 3.3. Economic Analyses

Scenario-based economic outcomes derived from graded replacement of SBM with BSFLM are summarized in Table 6. Under Scenario 1, protein meal cost (SBM + BSFLM) and total feed cost per quail decreased progressively with increasing BSFLM inclusion, resulting in a reduction in feed cost per kg live weight from 48.08 TL/kg at 0% replacement to 39.28 TL/kg at 100% replacement. In Scenario 2, protein meal cost showed minor variation across treatments, whereas total feed cost per quail was lowest at the 50% replacement level, corresponding to the minimum feed cost per kg live weight (44.61 TL/kg). In Scenario 3, both protein meal cost and total feed cost increased with increasing BSFLM inclusion, leading to higher feed cost per kg live weight at 75% and 100% replacement compared with the control. Across all scenarios, feed cost per kg live weight ranged between 39.28 and 51.92 TL/kg, reflecting scenario-dependent shifts in economic outcomes associated with BSFLM inclusion level.

## 4. Discussion

In the present study, the replacement of SBM with BSFLM resulted in a consistent biological response in Japanese quails, characterized by the maintenance of body weight across all ages alongside improvements in feed utilization efficiency at intermediate inclusion levels. The absence of statistically significant differences in body weight at days 28, 35, and 42 indicates that BSFLM was able to adequately meet the birds’ amino acid and energy requirements throughout the growing period, even when included at high dietary proportions. This finding supports previous evidence demonstrating that BSFLM possesses a nutritionally balanced amino acid profile and sufficient metabolizable energy to sustain growth performance when diets are properly formulated [3,22].

Although cumulative feed intake did not differ among treatments during the early growth phase, significant reductions were observed at days 35 and 42, particularly in quails receiving the 50% BSFLM diet. This reduction in feed intake, combined with a concomitant improvement in feed conversion ratio (FCR) at day 42, suggests that dietary BSFLM influenced growth performance primarily through improved feed efficiency, rather than increased feed consumption. Similar response patterns have been reported in quails and broiler chickens fed BSFLM-based diets, where feed efficiency improvements occurred without proportional increases in body weight or feed intake, especially at moderate inclusion levels [10,11,14].

The superior feed efficiency observed at the intermediate inclusion level may reflect a nutritional balance in which the high-quality protein and lipid fractions of BSFLM are effectively utilized, while potential constraints associated with higher chitin intake remain below critical thresholds. Chitin, a structural polysaccharide present in insect exoskeletons, has been widely recognized as a factor that may limit nutrient digestibility at elevated inclusion levels, potentially affecting intestinal morphology and digestive enzyme activity [3,11]. Recent evidence further indicates that the nutritional value and digestibility of BSFLM are strongly influenced by larval rearing substrate and processing conditions, which modulate protein quality, lipid composition, and chitin concentration [6,23]. In this context, the lack of linear or quadratic dose–response effects observed in the present study suggests that the biological system of quails was able to accommodate a wide range of BSFLM inclusion levels without pronounced performance penalties.

Evidence from broiler quails and broiler chickens corroborates these findings, indicating that partial replacement of conventional protein sources with BSFLM does not impair growth performance or feed efficiency when inclusion levels remain within biologically tolerable limits [9,14,24]. Moreover, recent meta-analytical evaluations have demonstrated that BSFLM inclusion at low to moderate levels does not compromise overall growth performance in poultry, while responses at higher inclusion rates are variable and dependent on factors such as diet formulation, processing method, and bird genotype [4,12,13,25]. The absence of treatment effects on mortality in the present study further supports the safety of BSFLM inclusion in quail diets, consistent with previous experimental reports [11,14,26].

To the best of our knowledge, limited information is available regarding the effects of BSFLM on growth-curve descriptors in poultry, as most studies primarily report conventional performance traits such as body weight, feed intake, and feed conversion ratio. The absence of treatment effects on Gompertz parameters, including mature weight, growth rate, and inflection-point traits, indicates that BSFLM inclusion did not alter the birds’ intrinsic growth trajectory. Instead, growth patterns remained biologically stable across dietary treatments, suggesting that the genetically determined growth potential of quails was preserved. This observation aligns with broader evidence indicating that moderate inclusion of insect-derived protein sources supports growth without disrupting inherent growth dynamics [12]. Furthermore, the magnitude of the estimated Gompertz parameters and inflection-point characteristics in this study closely matches those previously reported for Japanese quail populations, reinforcing the biological plausibility of the observed growth responses [27].

The stepwise incorporation of BSFLM into quail diets did not significantly influence carcass yield, cold carcass weight, major carcass components, or abdominal fat deposition. The stability of carcass traits observed across treatments indicates that nutrient partitioning and energy allocation were not adversely affected by dietary BSFLM inclusion. Comparable findings have been reported in quails and broiler chickens, where carcass yield and breast muscle development remained unchanged despite substantial replacement of conventional protein sources with BSFL-based meals [10,15,25]. Meta-analytical evidence further confirms that moderate levels of BSFLM supplementation do not induce significant changes in carcass traits, supporting the robustness of insect-derived proteins as alternatives to SBM in poultry nutrition [12,13].

The improvement in feed conversion ratio observed at the 50% replacement level may reflect a nutritional balance in which the protein and lipid fractions of BSFLM are aligned with the birds’ growth requirements, while potential constraints associated with higher inclusion levels remain below critical thresholds. At intermediate inclusion, BSFLM may support more efficient feed use through a combination of adequate amino acid supply ensured by formulation strategy, the contribution of dietary lipids to metabolizable energy, and the functional effects of moderate chitin intake. However, a limitation of the present study is the absence of direct measurements of nutrient and amino acid digestibility. Therefore, improvements in feed conversion observed at intermediate inclusion levels should be interpreted as indicators of enhanced feed efficiency rather than definitive evidence of increased nutrient digestibility. Future research incorporating digestibility assays would be valuable for elucidating the underlying mechanisms.

The economic relevance of BSFLM as a partial substitute for SBM must be interpreted within a broader price-risk and currency-sensitivity framework, particularly under conditions characteristic of import-dependent feed markets such as Turkey. In the present study, the integration of cost efficiency indices, marginal substitution analysis, and scenario-based sensitivity testing revealed that the economic response to BSFLM inclusion is governed not only by absolute ingredient prices but also by the interaction between biological feed efficiency and exposure to exchange-rate volatility [28,29,30].

Cost efficiency indices expressed on an output basis (feed cost per kg live weight) demonstrated that intermediate BSFLM inclusion, notably at 50% replacement, consistently aligned biological efficiency gains with economically resilient outcomes across contrasting price scenarios [18]. This pattern is critical because improvements in feed conversion and cumulative feed intake at intermediate inclusion levels translated into lower or stabilized unit production costs, even when total feed cost per bird did not decrease monotonically. The absence of changes in Gompertz growth parameters further indicates that these efficiency gains occurred within a biologically stable growth framework, reinforcing their relevance for commercial predictability rather than short-term performance distortion.

Marginal substitution and break-even analyses highlighted that economic neutrality between SBM and BSFLM is achieved within finite and realistic BSFLM price thresholds when biological efficiency gains are accounted for. This finding challenges simplistic price-based adoption criteria and emphasizes that BSFLM competitiveness should be evaluated against efficiency-adjusted cost benchmarks rather than ingredient prices alone. In practice, this means that BSFLM does not need to be unequivocally cheaper than SBM to remain economically viable, provided that feed efficiency advantages persist [31].

The scenario-based sensitivity analysis gains particular significance in the Turkish context, where both SBM and BSFLM costs are directly or indirectly sensitive to exchange-rate movements. SBM, as a globally traded commodity priced in foreign currency, exhibits high and rapid exchange-rate pass-through to domestic feed prices due to Turkey’s net importer status. In contrast, BSFLM price formation differs fundamentally across scenarios. While the high-cost scenario (S3), derived from European production benchmarks, is likewise sensitive to Euro-denominated cost structures, local and waste-based BSFLM production models (S1 and partially S2) rely more heavily on domestic inputs and are therefore less exposed to immediate currency shocks. This asymmetry in exchange-rate transmission confers BSFLM a comparatively more stable cost profile, even under conditions where its absolute price advantage over SBM is limited. In this regard, BSFLM may be conceptualized not merely as an alternative protein ingredient but as a buffer protein source capable of dampening feed-cost volatility associated with imported protein meals [32]. This buffering function is particularly relevant given Turkey’s substantial organic waste potential. According to national waste statistics, approximately 120 thousand tons of compostable organic municipal waste were collected in 2024 alone, excluding agricultural by-products and food-industry residues. The valorization of such locally available substrates for BSFL production extends the economic benefits beyond feed formulation by reducing waste management costs [32], strengthening local value chains, and mitigating dependence on imported protein sources [28,33].

## 5. Conclusions

The replacement of SBM with BSFLM in Japanese quail diets was not associated with detrimental effects on growth performance, survivability, or carcass characteristics. The maintenance of body weight and carcass yield, together with the improvement in feed conversion ratio observed at intermediate inclusion levels, indicates that BSFLM can support efficient feed use without disrupting overall growth dynamics. The absence of treatment effects on Gompertz growth-curve parameters further confirms that the birds’ intrinsic growth trajectory remained biologically stable, suggesting that dietary BSFLM did not interfere with genetically determined growth patterns. In addition, the absence of differences in carcass composition and abdominal fat deposition among dietary groups indicates that energy partitioning was not adversely affected by the feeding treatments. From an economic perspective, BSFLM inclusion generated a dual effect by improving feed efficiency at the production level while reducing exposure to exchange-rate-driven cost volatility. Given Turkey’s strong dependence on imported SBM, these findings suggest that the economic value of BSFLM lies primarily in strategic partial substitution rather than complete replacement, particularly where biological efficiency gains can be leveraged alongside locally based production and organic waste valorization. Under conditions of increasing uncertainty in global feed markets, moderate BSFLM inclusion emerges as a practical and risk-aware feeding strategy that aligns production efficiency with economic resilience and supply-chain stability.

## Figures and Tables

**Table 1:** Composition and analyzed values (% diet) of feeds incorporating varying percentages of BSFLM.

Ingredients (%)	Experimental Diets				
	BSF0	BSF25	BSF50	BSF75	BSF100
Yellow corn	50.1	50.2	51.2	51.8	52.1
Soybean meal	36	27	18	9	0
BSFL meal	0	9	18	27	36
Meat-bone meal	4.8	4.7	4.3	4.1	4.0
Sunflower oil	5.1	5.0	4.3	4.1	3.9
Dicalcium phosphate	0.52	0.80	0.82	0.83	0.85
Calcium carbonate	1.5	1.3	1.2	1.1	1.0
Sodium chloride	0.22	0.22	0.22	0.22	0.22
Sodium bicarbonate	0.12	0.12	0.12	0.12	0.12
DL-Methionine	0.18	0.21	0.24	0.26	0.28
L-Lysine	0.49	0.50	0.53	0.55	0.57
Threonine	0.21	0.21	0.21	0.21	0.21
Vitamin–mineral premix ^1^	0.60	0.60	0.60	0.60	0.60
Choline chloride	0.02	0.02	0.02	0.02	0.02
3-Phytase	0.10	0.10	0.10	0.10	0.10
Total	100	100	100	100	100
Analyzed composition					
ME, kcal/kg diet	2991	3008	2995	3011	3003
CP	24.1	24.1	24.0	24.1	24.2
EE	7.4	7.4	7.4	7.4	7.4
CF	3.0	3.2	3.3	3.4	3.4
Calcium	0.98	1.03	1.08	1.07	1.11
Phosphorus	0.49	0.53	0.52	0.55	0.53
Lysine digestible	1.2	1.2	1.2	1.2	1.2
Methionine digestible	0.81	0.81	0.81	0.81	0.81
Threonine digestible	0.62	0.62	0.62	0.62	0.62

ME metabolizable energy, CP crude protein, EE ether extract, CF crude fiber, DM dry matter. Minor deviations from 1000 g/kg were due to rounding during diet formulation. ^1^ The vitamin–mineral premix provides the following per kilogram of diet: Retinyl acetate 9500 IU, Cholecalciferol 4500 IU, α-tocopheryl acetate 25 IU, Menadione 3.0 mg, Vitamin B1 3.0 mg, Vitamin B2 7 mg, Vitamin B6 3.50 mg, Vitamin B12 0.50 mg, Calcium Pantothenate 10.0 mg, Nicotinamide 50.0 mg, Folic Acid 1.80 mg, Biotin 0.225 mg, Copper 10 mg, Iron 90 mg, Zinc 100 mg, Manganese 100 mg, Iodine 2.50 mg, Selenium 0.35 mg, Choline 5 g.

**Table 2:** Expression and point of inflection coordinates of Gompertz growth function.

Growth Model	Equation	IPA	IPW
Gompertz	$Y_{t}=\beta_{0}\cdot e^{{-\beta }_{1}e^{-\beta_{2}t}}$	$ln\left(\beta_{1}\right)/\beta_{2}$	$\beta_{0}/e$

**Table 3:** The mean values of some performance traits and results of variance analyses.

Trait	Treatment (SBM Replacement with BSFLM, %)					SEM	*p* Value	Responses to BSFLM	
	0	25	50	75	100			Linear	Quadratic
BW28 (g)	151.20	153.95	153.62	154.36	152.04	0.80	0.693	0.705	0.841
BW35 (g)	181.63	184.15	186.10	184.30	186.55	1.08	0.641	0.655	0.701
BW42 (g)	201.39	201.69	206.60	204.47	204.06	1.19	0.097	0.749	0.894
FI28 (g)	327.88	322.27	321.19	323.12	325.57	1.92	0.596	0.843	0.887
FI35 (g)	473.03 ^a^	465.01 ^ab^	456.45 ^c^	469.42 ^a^	461.97 ^bc^	2.83	0.044	0.645	0.789
FI42 (g)	661.20 ^a^	648.68 ^b^	637.26 ^c^	648.92 ^b^	651.46 ^b^	3.86	0.018	0.551	0.612
FCR28 (g/g)	2.12	2.10	2.11	2.11	2.15	0.02	0.914	0.943	0.964
FCR35 (g/g)	2.63	2.54	2.48	2.57	2.50	0.02	0.134	0.438	0.687
FCR42 (g/g)	3.31 ^a^	3.20 ^b^	3.13 ^c^	3.22 ^b^	3.22 ^b^	0.02	0.020	0.105	0.703
Mortality (%)	11.67 (7/60)	3.33 (2/60)	5.00 (3/60)	5.00 (3/60)	6.67 (4/60)	1.41	0.089	0.475	0.583

HW: Hatching weight; BW: Body weight at 28th, 35th, 42nd days; FI: Cumulative feed intake at 28th, 35th, 42nd days; FCR: Cumulative feed conversion ratio at 28th, 35th, 42nd days; SEM: Standard error of mean. Values are means of 60 birds per treatment (*n* = 60). ^a–c^ Means within the same row with different superscript letters differ significantly (*p* < 0.05).

**Table 4:** The mean values of parameters of Gompertz growth curve and results of variance analyses.

Trait	Treatment (SBM Replacement with BSFLM, %)					SEM	*p* Value	Responses to BSFLM	
	0	25	50	75	100			Linear	Quadratic
β_0_	246.33	244.93	252.96	248.82	252.60	2.13	0.676	0.709	0.856
β_1_	3.30	3.36	3.32	3.31	3.33	0.02	0.825	0.888	0.904
β_2_	0.070	0.072	0.069	0.071	0.068	0.001	0.509	0.611	0.748
IPW	90.63	90.11	93.07	91.54	92.94	0.78	0.676	0.758	0.803
IPA	17.41	17.15	17.56	17.26	17.78	0.15	0.683	0.845	0.911

β_0_: Mature weight parameter; β_1_: Shape parameter; β_2_: Instantaneous growth rate parameter; IPW: Body weight at inflection point of growth curve; IPA: Age at inflection point of growth curve; SEM: Standard error of mean. Values are means of 60 birds per treatment (*n* = 60).

**Table 5:** The mean values of slaughter–carcass characteristics and results of variance analyses.

Trait	Treatment (SBM Replacement with BSFL, %)					SEM	*p* Value	Responses to BSFLM	
	0	25	50	75	100			Linear	Quadratic
Carcass yield (%)	69.50	69.84	69.78	69.71	69.58	0.16	0.960	0.975	0.984
Cold carcass (g)	140.02	141.02	144.21	142.34	141.97	0.89	0.656	0.742	0.888
Breast (g)	55.21	55.15	56.12	55.29	54.95	0.38	0.885	0.905	0.952
Leg (g)	29.93	30.53	30.77	30.91	30.59	0.19	0.576	0.738	0.854
Wing (g)	11.06	11.10	11.52	11.69	11.58	0.10	0.130	0.425	0.555
Abd. fat (g)	1.03	1.02	0.96	1.12	0.92	0.03	0.267	0.502	0.704

Abd. Fat: Abdominal fat pad; SEM: Standard error of mean. Values are means of 30 birds per treatment (15 males and 15 females; *n* = 30).

**Table 6:** Scenario-based protein meal (SBM + BSFLM) cost, total feed cost, and feed cost per kg live weight at 42 d under graded replacement of SBM with BSFLM.

	Treatment (SBM Replacement with BSFLM, %)				
Variable	0	25	50	75	100
Scenario 1 (SBM 17.1; BSFLM 10.6)					
Meal cost (TL/quail)	4.07	3.61	3.18	2.86	2.49
Feed cost (TL/quail)	9.68	9.12	8.59	8.36	8.02
Feed cost/kg BW (TL/kg)	48.08	45.22	41.56	40.9	39.28
Scenario 2 (SBM 17.1; BSFLM 16.1)					
Meal cost (TL/quail)	4.07	3.93	3.81	3.82	3.78
Feed cost (TL/quail)	9.68	9.44	9.22	9.33	9.31
Feed cost/kg BW (TL/kg)	48.08	46.81	44.61	45.62	45.6
Scenario 3 (SBM 17.1; BSFLM 21.6)					
Meal cost (TL/quail)	4.07	4.26	4.44	4.78	5.07
Feed cost (TL/quail)	9.68	9.76	9.85	10.29	10.6
Feed cost/kg BW (TL/kg)	48.08	48.4	47.67	50.33	51.92

The soybean meal (SBM) price (17.1 TL/kg) represents the arithmetic mean of official spot and registration prices reported by multiple regional commodity exchanges in Turkey, reflecting domestic market conditions.

## Data Availability

All data generated or analyzed during this study are included in this published article. The datasets used and/or analyzed in this study are available from the corresponding author upon reasonable request.
